# Mercury pollution for marine environment at Farwa Island, Libya

**DOI:** 10.1186/s40201-016-0246-y

**Published:** 2016-02-20

**Authors:** Adel A.S. Banana, R. M. S. Radin Mohamed, A. A. S. Al-Gheethi

**Affiliations:** Environment Engineering Department, Subrata College, University of Zawia, Zawia, Libya; Faculty of Civil & Environment Engineering, UTHM, Parit Raja, Malaysia; High Institute of health sciences, Sana’a, Yemen

**Keywords:** Mercury, Fishes, Contamination, Farwa Island

## Abstract

**Background:**

Farwa is an Island in Libya receives petrochemical wastes generated from General Company of Chemical Industries (GCCI) since more than 40 years.

**Aim:**

The present work aimed to determine the concentrations of mercury (Hg^+2^) in fish, marine plants and sediment collected from Farwa lagoon to evaluate effect of industrial wastewater from GCCI on the marine environment.

**Methods:**

Hundred and twelve samples of fish, pearl oyster, cuttlefish sediments and marine plants were analyzed to determine Hg^2+^ concentration during the period from January to August 2014 by using Atomic Absorption Spectrometer (AAS).

**Results:**

The highest concentration of Hg^2+^ was detected in *Pinctada radiata* (11.67 ± 3.30 μgg^**−**1^) followed by *Serranus scriba* (6.37 ± 0.11 μg g^**−**1^) and *Epinephelus marginatus* (6.19 ± 0.02 μg g^**−**1^). About 75 % of marine plants contained the maximum contaminations during the summer season. In fish samples Hg^2+^ concentrations exceeded the levels provided by international standards.

**Conclusions:**

The fish at Farwa lagoon is heavily contaminated with Hg^2+^ which may represent a source for mercury poisoning for human.

## Background

The increasing of industrial activities has led to increase the contamination of environment with several types of pollutants as due to discharge of industrial wastewater into the environment and aquatic system. petrochemicals factors is among various of industrials process which produce heavily contaminated wastewater. In the developed countries the wastewater are treated using advanced technologies such as reverse osmosis, nanotube carbon, adsorption process using different types of adsorbents as well as photo-degradation processes of degradable toxic compounds. Those technologies have high efficiency to remove most toxic substances from wastewater before final disposal into the environment. Others technologies such as multi-walled carbon nanotube/tungsten oxide (MWCNT/WO_3_) and alumina nano-particles polyamide membrane still under investigation and they exhibited high efficiency for the removal and degrade various types of pollutants based on the lab scale experiments [1–7].

In the term of heavy metals contamination, the petrochemical industries represent one of the main sources for generation of these toxics into the environment. the adsorption process materials is the most common treatment process to remove heavy metals from wastewater. Recently, some authors focused on improvement this process to be high efficiency. Gupta et al. [8] has combined the magnetic properties of iron oxide with adsorption properties of carbon nanotubes to increase the removal of Cr^2+^ ions.

Heavy metals are groups of elements with high molecular weights that are not degraded when taken into the body; instead, they accumulate in specific body organs and cause illness. Heavy metals have the potential to disrupt the metabolism and biological activities of many proteins because it can oxidize the sulfhydryl groups [9]. Among several of heavy metals, mercury (Hg^2+^) is the most toxic element for organisms [10–12]. Hg^2+^ is very toxic pollutant that contaminates fish around the world, therefore fish represent the main source of Hg^2+^ for human [13]. The studies indicated that mercury accumulation in the oceans correlates with the rising tide of mercury pollution. The most serious Hg^2+^ poisoning has been occurred due to consumption of Hg^2+^ contaminated fish and other seafood polluted by industrial wastewater [14]. However, information for mercury contamination of fishes and marine environment in Libya is unavailable; this might due to absence of academic research for more than 40 years. Therefore, the present work aimed to evaluate the concentrations of Hg^2+^ in fish, sea woods and sediments at Farwa Island, Libya that received petrochemical wastes generated from General Company of Chemical Industries (GCCI) for more than 40 years.

## Methods

### Study area

**Fig. 1:**
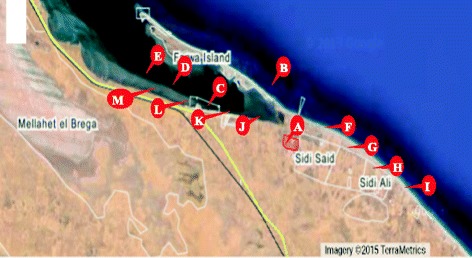
Google map of study area, A) GCCI B) site of fish samples collection C) site of marine plant samples collection (100 m); D) site of marine plant samples collection (1000 m); E) site of marine plant samples collection (3000 m); F) site of sediment samples collection (100 m, W); G) site of sediment samples collection (500 m, W); H) site of sediment samples collection (1000 m, W); I) site of sediment samples collection (3000 m, W); J) site of sediment samples collection (100 m, E); K) site of sediment samples collection (500 m, E); L) site of sediment samples collection (1000 m, E); site of sediment samples collection (3000 m, E)

Farwa Island is located on the Mediterranean in West Zawya, Libya (33° 04’ N, 1° 50’ E to 33° 08’ N and 11° 32’ E) from Abu- Kamash east to the Tunisian border in the west (Fig. 1). It comprises Farwa lagoon that covering an area of 32 km^2^ and is the largest lagoon on the Libyan coast. GCCI is located at Abu- Kamash chemical complex. GCCI was opened in 1970s and consist of 3 units that produce 104,000 tonnes/year Ethylene di-chloride, 60, 000 tonnes poly vinyl chloride (PVC), 50,000 tonnes caustic soda and 45,000 tonnes chlorine. In addition to sodium carbonate, sodium hypochlorite and HCl. GCCI has four dumping sites, two of them are located on the west while another two are located on the east.

### Collection and analysis of samples

Hundred ninety two samples (in triplicate, 3 sample/month) of fishes, oysters, cuttlefish, magnoliophyta plants and sediments were collected from marine environment around of Farwa Island, Libya during the period from January to August 2014. The marine organisms collected samples included ten types of fishes, only one type of oyster and one type of cuttlefish. These samples were collected using local fishermen. Magnoliophyta plant samples were collected from different location around Farwa Lagoon, Zone I (100 m), Zone II (1000 m) and Zone III (3000 m). These locations represent the distance between the sampling point and the factory and they were selected because its very close to GCCI and the possibility to heavy contamination with Hg^2+^ is high. The samples were transported inside ice box to the laboratory and kept in deep freezer at −20 °C until analysis.

Sample preparation and analysis were carried out according to Bernhard [15]. Liver, muscle, gill, heart, air sac and stomach-intestine were removed before the analysis [16]. Fish samples were homogenized in a blender. Magnoliophyta plants were cut out into small pieces (5 mm in diameter) and then homogenized in a blender. A weight of 10 g of homogenate for each of fish and magnoliophyta plants was digested according to APHA [17]. In briefly; five mL of HNO_3_ (65 %) and 5 mL of H_2_SO_4_ were added into sample placed inside flask (100 mL). The mixture was heated on a hot plate (70–80 °C) for 30 min to the lowest volume (20 mL) before precipitation occurs. The digestion step was continued until light colored, clear solution was observed. The flask walls was washed with distilled water and filtered using Whatman, 125 mm Ø, filter papers (Cat No. 1001 England). The filtrate was transported into a volumetric flask (100 mL) with 10 mL water and mixed thoroughly.

Sediment samples (1 kg) were collected by using grab sampler from eight sites located on the west and east of GCCI. Samples were transported to the laboratory and dried in oven at 50 °C. After that, sediment samples were powdered and passed through 160 μm sieve. The samples packed in paper bags and stored in deep freezer at −20 °C prior to analysis. The mercury was extracted from the samples with 10 mL HNO_3_/HCl (1:3 v/v) by using a microwave digestion system as described above.

The Hg^2+^ concentrations in the digested samples were determined by an atomic absorption spectrophotometer (AAS) (Model P.E.A ANALYST 100, HGA-800 and MHS-10, Perkin Elmer, USA).

The concentrations of heavy metals was calculated (μg g^−1^) using Eqs. (1)

$$ MetalConcentration=A\times B/C $$

Where

*A = concentrations of metals in digested solution μg g*^*−1*^

*B = final volume of digested solution mL*

*C = sample size, gram*

### Data analysis

The data were not normally distributed, therefore, they were log transformed and subjected to parametric statistics. The differences in Hg^2+^ concentrations of samples investigated were tested by ANOVA. The statistical analyses was performed SPSS (version 11.5).

## Results and discussion

**Fig. 2:**
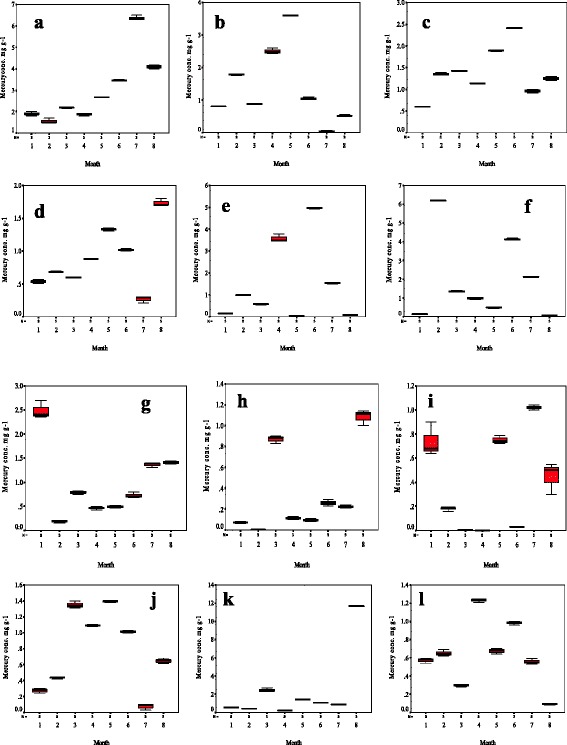
Seasonal distribution of Hg^2+^ concentrations in fish, oysters and cuttlefish samples collected from Farwa lagoon, Libya; **a**) *Serranus scriba;* **b**) *Oedalechilus labeo,* **c**) *Diplodus vulgaris;* **d**) *Dicentrarchus labrax;* **e**) *Lithognathus mormyrus;* **f**) *Epinephelus marginatus;* **g**) *Sarpa salpa;* **h**) *Sciaena umbra;* **i**) *Pagrus pagrus;* **j**) *Caranx crysos;* **k**) *Pinctada radiate;* **l**) *Sepia officinalis*

The present study investigated the mercury contamination of marine environment included fishes, oysters, cuttlefish, magnoliophyta plants and sediments at Farwa Island, Libya that are received industrial wastewater generated from GCCI since 40 years ago. The concentration of mercury at this place has not reported before, thus the current work was conducted to evaluate the effect of petrochemical wastewater on the environment. The results revealed that the Hg^2+^ concentration differed significantly (p < 0.05) during the period of study (Table 1). These variables may be due to the climatic conditions of the area, winter season extends from November to March and is generally cold and rainy with unstable winds blowing from different directions which lead to cause dilution of Farwa lagoon, while summer season (May to September) is rather hot and dry [18]. The mean of Hg^2+^ concentrations in fish, oysters and cuttlefish samples collected during the period study are presented in Table 2. It can be noted that the Hg^2+^ concentrations ranged from 3.13 ± 1.5 μg g^−1^ in *Serranus scriba* to 0.34 ± 0.33 μg g^−1^ in *Sciaena umbra.* The distributions of Hg^2+^ concentrations for each species in the period from January to August 2014 are depicted in Fig. 2. It shown that the highest concentration of Hg^2+^ was detected in *Pinctada radiata* (11.67 ± 3.30 μgg^−1^) in August, followed by *Serranus scriba* (6.37 ± 0.11 μg g^−1^) in July and *Epinephelus marginatus* (6.19 ± 0.02 μg g^−1^) in February. The *Serranus scriba* have high concentration of Hg^2+^ during the study period from January to July followed by *Epinephelus marginatus*, the average was 2.83 *vs.* 2.18 μg g-1. The lowest Hg^2+^ concentrations were detected in *Pagrus pagrus* (0.001 μg g^−1^) and *Sciaena umbra* (0.01 μg g^−1^). Both types contained the lowest average concentrations during the period of study (0.33 and 0.36 μg g^−1^ respectively). *Lithognathus mormyrus* has the highest Hg (3.59 ± 0.19 μg g^−1^) among the fish samples collected in April, whereas *Oedalechilus labeo* has the highest Hg (3.59 ± 0.01 μg g^−1^) among the fish samples collected in May. In June, the highest Hg^2+^ was determined in *Lithognathus mormyrus* (4.97 ± 0.04 μg g^−1^).

**Table 1:** ANOVA Analysis of Hg^2+^ concentrations in different fish samples during the period of study from January to August 2014

Sample		Sum of Squares	df	Mean Square	F	Sig.
*Serranus scriba*	Between groups	54.369	7	7.767	1257.814	.000
	Within groups	.099	16	.006		
	Total	54.468	23			
Oedalechilus labeo	Between groups	28.478	7	4.068	2697.193	.000
	Within groups	.024	16	.002		
	Total	28.502	23			
*Diplodus vulgaris*	Between groups	6.588	7	.941	1146.640	.000
	Within groups	.013	16	.001		
	Total	6.602	23			
*Dicentrarchus labrax*	Between groups	4.697	7	.671	712.616	.000
	Within groups	.015	16	.001		
	Total	4.712	23			
*Lithognathus mormyrus*	Between groups	70.353	7	10.050	2138.378	.000
	Within groups	.075	16	.005		
	Total	70.428	23			
*Epinephelus marginatus*	Between groups	99.308	7	14.187	16690.375	.000
	Within groups	.014	16	.001		
	Total	99.321	23			
*Sarpa salpa*	Between groups	11.577	7	1.654	301.838	.000
	Within groups	.088	16	.005		
	Total	11.664	23			
*Sciaena umbra*	Between groups	3.448	7	.493	467.260	.000
	Within groups	.017	16	.001		
	Total	3.465	23			
*Pagrus pagrus*	Between groups	3.373	7	.482	98.591	.000
	Within groups	.078	16	.005		
	Total	3.451	23			
*Caranx crysos*	Between groups	5.237	7	.748	949.947	.000
	Within groups	.013	16	.001		
	Total	5.249	23			
*Pinctada radiata*	Between groups	310.444	7	44.349	6809.846	.000
	Within groups	.104	16	.007		
	Total	310.548	23			
*Sepia officinalis*	Between groups	2.703	7	.386	639.161	.000
	Within groups	.010	16	.001		
	Total	2.713	23

**Table 2:** Hg^2+^ concentrations in Fishes collected from Farwa lagoon, Libya which received petrochemical wastes from General Company of Chemical Industries (GCCI), (±SD represent the standard division from the mean, *n* = 24 for each sample)

Sample No.	Family name	English name	Science name	Hg concentration (μg g^−1^)
*1*	*Serranidae*	*Painted comber*	*Serranus scriba*	*3.13 ± 1.5*
*2*	*Mugilidae*	*Boxlip Mullet*	*Oedalechilus labeo*	*1.4 ± 1.1*
*3*	*Sparidae*	Common *Two*-*Banded Seabream*	*Diplodus vulgaris*	1.4 *± 0.53*
*4*	*Moronidae*	*European seabass*	*Dicentrarchus labrax*	*0.89 ± 0.45*
*5*	*Sparidae*	*Striped sea bream*	*Lithognathus mormyrus*	1.5 *± 0.7*
*6*	*Serranidae*	*Dusky Grouper*	*Epinephelus marginatus*	*1.9 ± 2.0*
*7*	*Sparidae*	*Salema*	*Sarpa salpa*	*0.99 ± 0.71*
8	Sciaenidae	*sculpin*	*Sciaena umbra*	*0.34 ± 0.33*
*9*	*Dentex macrophthalmus*	*Red porgy*	*Pagrus pagrus*	*0.39 ± 0.38*
*10*	*Carangidae*	*blue runner*	*Caranx crysos*	*0.78 ± 0.48*
*11*	*Oyster*	*Rayed Pearl Oyster*	*Pinctada radiata*	*2.3 ± 3.6*
*12*	*Cuttlefish*	*common cuttlefish*	*Sepia officinalis*	*0.63 ± 0.34*

The analysis for association between Hg^2+^ concentrations in fish, oysters as well as cuttlefish samples and months indicated that the concentration of Hg^2+^ in *Serranus scriba, Dicentrarchus labrax, Sciaena umbra* and *Pinctada radiata* associated significantly (*p* < 0.05) to the seasons with R^2^ 0.64, 0.24, 0.21 and 0.34 respectively (Table 3). The Hg^2+^ concentrations in magnoliophyta plants are presented in Table 4. It can be noted that the maximum concentration was detected in the samples collected from place near of GCCI (100 m). The highest concentrations were determined in samples collected during April, where, 2.33 ± 0.60, 1.44 ± 0.42 and 0.96 ± 0.12 μg g^−1^ were determined in samples collected from zone I, II and III respectively. The lowest Hg^2+^ was recorded in samples collected from zone III (0.02 ± 0.00 μg g^−1^) during January. In comparison with the study conducted by Pergent-Martini [19] which was carried out on the mercury contamination in the *Posidonia oceanica* Collected from mediterranean sea. It can be noted that the Hg^2+^ concentrations in this study was quite high. There would be due to dispose of wastewater generated from GCCI into the sea without treatment process since 40 years ago.

**Table 3:** Measures of association between Hg^2+^ concentrations in fish, *oysters as well as cuttlefish* samples and months

Sample*month	R	R Squared	Eta	Eta Squared	Significance (p value)
*Serranus scriba*	0.80	0.64	0.99	0.99	0.01
*Oedalechilus labeo*	−0.228	0.05	1.00	0.99	0.14
*Diplodus vulgaris*	0.327	0.11	0.99	0.99	0.06
*Dicentrarchus labrax*	0.493	0.24	0.99	0.99	0.01
*Lithognathus mormyrus*	0.191	0.04	0.99	0.99	0.18
*Epinephelus marginatus*	−0.173	0.03	1.00	1.00	0.21
*Sarpa salpa*	−0.068	0.01	0.99	0.99	0.38
*Sciaena umbra*	0.454	0.21	0.99	0.99	0.01
*Pagrus pagrus*	0.215	0.05	0.98	0.98	0.16
*Caranx crysos*	0.006	0.00	0.99	0.99	0.45
*Pinctada radiata*	0.587	0.34	1.00	1.00	0.01
*Sepia officinalis*	−0.188	0.04	0.99	0.99	0.19

**Table 4:** Hg^2+^ concentrations in magnoliophyta plant samples collected from different distance of GCCI at Farwa lagoon, Libya (±SD represent the standard division from the mean, *n* = 3 for each sample per month)

Sample/month	Hg^2+^ concentrations (μg g^−1^)		
	Zone I (100 m)	Zone II (1000 m)	Zone III (3000 m)
1	0.82 ± 0.20	0.93 ± 0.13	0.02 ± 0.00
2	0.39 ± 0.12	0.50 ± 0.08	0.10 ± 0.09
3	2.10 ± 0.91	0.57 ± 0.16	0.82 ± 0.20
4	2.33 ± 0.60	1.44 ± 0.42	0.96 ± 0.12
5	1.06 ± 0.18	0.79 ± 0.12	0.75 ± 0.19
6	1.00 ± 0.13	1.38 ± 0.92	0.54 ± 0.20
7	0.87 ± 0.30	0.25 ± 0.09	0.64 ± 0.21
8	1.40 ± 0.42	1.07 ± 0.13	0.71 ± 0.15

The present study revealed that the concentrations of Hg^2+^ in all types of fish samples were more than the standards limits recommended by FDA and FAO-WHO [20, 21]. According to U.S. EPA [22], Hg^2+^ should be less than 0.3 μg g^−1^ wet fish muscle tissue for protection of human health. However, Zaza et al. [13] reported that the minimum level of Hg^2+^ is 0.5 μg g^−1^ for fish species. In the present study, the minimum concentration of Hg^2+^ was 1 μg g^−1^ in *Pagrus pagrus.* Fish consumption is one of the major factors of Hg^2+^ intake for humans [23, 24]. Hg^2+^ is very dangerous for pregnant woman because mercury is most harmful to developing foetuses, infants, and young children.

High Hg^2+^ concentration was detected in sediment samples collected from the West of GCCI than those collected from the East. However, both sites contain high concentration of Hg^2+^. The Hg^2+^ concentration decreased significantly (*p* < 0.05) as the site distance from GCCI, the maximum Hg^2+^ was noted in sediment samples taken from the west (100 m from GCCI) where 11.14 ± 4.11 μg g^−1^ was recorded in April 2014 (Table 5). Among the sediment samples collected from the east, the samples taken in June contain 4.67 ± 1.62 μg Hg^2+^ g^−1^. The pollution of environmental area around GCCI represent a serious problem due to that the surrounding areas are used for agricultural purpose such as for Grapes, olives and almonds. More than 1500 people are living around the GCCI.

**Table 5:** Hg^2+^ concentrations in sediment samples collected from the west and east GCCI during the period January to August 2014 (±SD represent the standard division from the mean, *n* = 3 for each sample per month)

Sediments sample/month	Hg^2+^ Concentration (μg g^−1^)								
	West of GCCI				East of GCCI				Control
	<100 m	500 m	1000 m	3000 m	100 m	500 m	1000 m	3000 m	Zwara city (20 km)
1	3.54 ± 1.10	1.38 ± 0.14	1.09 ± 0.04	0.64 ± 0.32	1.96 ± 1.01	0.55 ± 0.06	0.03 ± 0.0	0.01 ± 0.0	0.001 ± 0.0
2	6.74 ± 3.14	4.06 ± 1.81	1.07 ± 0.52	0.32 ± 0.19	1.55 ± 0.71	1.12 ± 0.24	0.75 ± 0.18	0.01 ± 0.0	0.001 ± 0.0
3	4.58 ± 1.43	3.04 ± 0.93	0.06 ± 0.09	0.02 ± 0.01	2.07 ± 0.91	0.75 ± 0.18	0.05 ± 0.01	0.001 ± 0.0	0.001 ± 0.0
4	11.14 ± 4.11	2.64 ± 0.83	1.95 ± 0.31	0.56 ± 0.08	3.65 ± 1.04	1.47 ± 0.91	0.01 ± 0.0	0.001 ± 0.0	0.001 ± 0.0
5	8.50 ± 2.84	5.07 ± 2.81	1.01 ± 0.41	0.75 ± 0.11	3.12 ± 1.11	0.06 ± 0.01	0.003 ± 0.0	0.001 ± 0.0	0.001 ± 0.0
6	2.46 ± 1.31	3.65 ± 1.71	0.95 ± 0.71	0.07 ± 0.22	4.67 ± 1.62	1.5 ± 0.46	0.001 ± 0.0	0.01 ± 0.0	0.003 ± 0.0
7	5.21 ± 2.28	4.06 ± 1.93	0.50 ± 0.01	0.43 ± 0.17	2.13 ± 0.98	0.04 ± 0.0	0.01 ± 0.0	0.002 ± 0.0	0.001 ± 0.0
8	1.56 ± 0.73	3.25 ± 0.88	0.36 ± 0.03	0.05 ± 0.0	3.17 ± 0.51	1.45 ± 0.29	0.003 ± 0.0	0.002 ± 0.0	0.001 ± 0.0

Farwa Island has high fishery production, but this Island had been exposed for heavy pollution due to GCCI for more than 40 years. Farwa Island is the most important coastal and marine site in western Libya, in terms of its high marine and coastal biodiversity based on several surveys and studies during the last years. However, no information was recorded according to mercury pollution. This region is characterized by an exceptional importance in terms of fish and artisanal fisheries, aquaculture, sea birds, sea grass meadows, land/seascape features and, above all, as one of the few regions in the Mediterranean to experience active tidal movements. In addition to some endangered species which makes it an important area for larva and juvenile protection. In the term of biodiversity, Farwa has many economically important species and certain endangered species are recognized [18].

In the term of toxic pollutants in industrial wastewater and their environmental impact and health effect, the sea water around of GCCI should be treated to remove of Hg^2+^ ions. Variety of biological and physico-chemical methods for wastewater treatment has been developed. Among those technologies, reverse osmosis, activated carbon, advanced oxidation, alumina-coated carbon nanotubes, tire derived carbons, porous carbon, carbon nanotubes and fullerene and CNT/magnesium oxide composite have exhibited high efficiency for removal of heavy metals from different aqueous solution [25–33].

## Conclusions

It can be concluded that the heavily contamination of fish, oysters as well as cuttlefish in marine environment around GCCI represent a main source for food poisoning among peoples living in this area. Therefore, the contaminated area should be treated to prevent health risk associated with mercury contamination.
